# Real time *ex vivo* chemosensitivity assay for pancreatic adenocarcinoma

**DOI:** 10.18632/oncotarget.28508

**Published:** 2023-09-15

**Authors:** Dae Won Kim, Francisca Beato, Youngchul Kim, Alexandra F. Tassielli, Ruifan Dai, Jason W. Denbo, Pamela J. Hodul, Mokenge P. Malafa, Jason B. Fleming

**Affiliations:** ^1^Department of Gastrointestinal Oncology, Moffitt Cancer Center, Tampa, FL 33612, USA; ^2^Department of Biostatistics and Bioinformatics, Moffitt Cancer Center, Tampa, FL 33612, USA; ^*^These authors contributed equally to this work

**Keywords:** pancreatic cancer, sensitivity assay, chemotherapy

## Abstract

Background: Patient-derived organoids (PDOs) and xenografts (PDXs) have been extensively studied for drug-screening. However, their usage is limited due to lengthy establishment time, high engraftment failure rates and different tumor microenvironment from original tumors. To overcome the limitations, we developed real time-live tissue sensitivity assay (RT-LTSA) using fresh tumor samples.

Methods: Tissue slices from resected pancreatic cancer samples were placed in 96-well plates, and the slices were treated with chemotherapeutic agents. The correlation between the chemo-sensitivity of tissue slices and each patient’s clinical outcome was analyzed.

Results: The viability and tumor microenvironment of the tissue slices are well-preserved over 5 days. The drug sensitivity assay results are available within 5 days after tissue collection. While all 4 patients who received RT-LTSA sensitive adjuvant regimens did not develop recurrence, 7 of 8 patients who received resistant adjuvant regimens developed recurrence. We observed significantly improved disease-free survival in the patients who received RT-LTSA sensitive adjuvant regimens (median: not reached versus 10.6 months, *P* = 0.02) compared with the patient who received resistant regimens. A significant negative correlation between RT-LTSA value and relapse-free survival was observed (Somer’s D: −0.58; *P* = 0.016).

Conclusions: RT-LTSA which maintains the tumor microenvironment and architecture as found in patients may reflect clinical outcome and could be used as a personalized strategy for pancreatic adenocarcinoma. Further, studies are warranted to verify the findings.

## INTRODUCTION

Pancreatic adenocarcinoma is the third leading cause of cancer-related death in the United States [1]. In 2023, the number of new cases and deaths of pancreatic cancer are estimated to be 64,050 and 50,550, respectively [1]. Surgical resection is considered as the only potentially curative approach. However, only a limited number of patients are able to seek surgical resection, and over 80% of surgically resected patients ultimately relapse [2]. Currently, advanced pancreatic adenocarcinoma is treated with fluorouracil or gemcitabine-based chemotherapy including FOLFIRINOX (leucovorin, fluorouracil, irinotecan and oxaliplatin), gemcitabine/nab-paclitaxel and gemcitabine single agent depending on performance status and comorbidity of each patient. However, pancreatic cancer is recalcitrant to first-line chemotherapy. The objective response rates are only 7–31%, and a majority of patients develop disease progression within 6 months [3, 4]. In addition, most of patients with pancreatic cancer receiving chemotherapy develop significant adverse events which may profoundly affect quality of life and require hospitalization and aggressive supportive treatment such as antibiotics, hematopoietic growth factors and blood transfusion [3]. Due to aggressive nature of disease and significant toxicity of current chemotherapy, selection of the most efficacious chemotherapy for each patient with advanced pancreatic cancer may improve patient outcome and quality of life. Currently, no predictive biomarkers are available for pancreatic cancer. Therefore, stable and reproducible methods and/or biomarkers predicting response to therapy are urgently needed for preventing patients from unnecessary toxicity without clinical benefit.

Previously, we reported that a unique *ex vivo* live tissue sensitivity assay (LTSA) using patient-derived xenografts (PDXs) could predict clinical response to therapeutic agents in patients with pancreatic adenocarcinoma [5]. However, there are several limitations for wide use of the PDX-based assay in pancreatic cancer due to lengthy establishment time, high engraftment failure rate and different tumor microenvironment from original tumors [6–9].

To overcome the major hurdles of the PDX-based assay, we developed real time LTSA (RT-LTSA) using fresh tumor samples. In this study, we report a reliable and reproducible RT-LTSA with resected fresh tumor samples to predict patients’ clinical response to chemotherapy in pancreatic cancer.

## RESULTS

### Establishment of RT-LTSA

**Figure 1:**
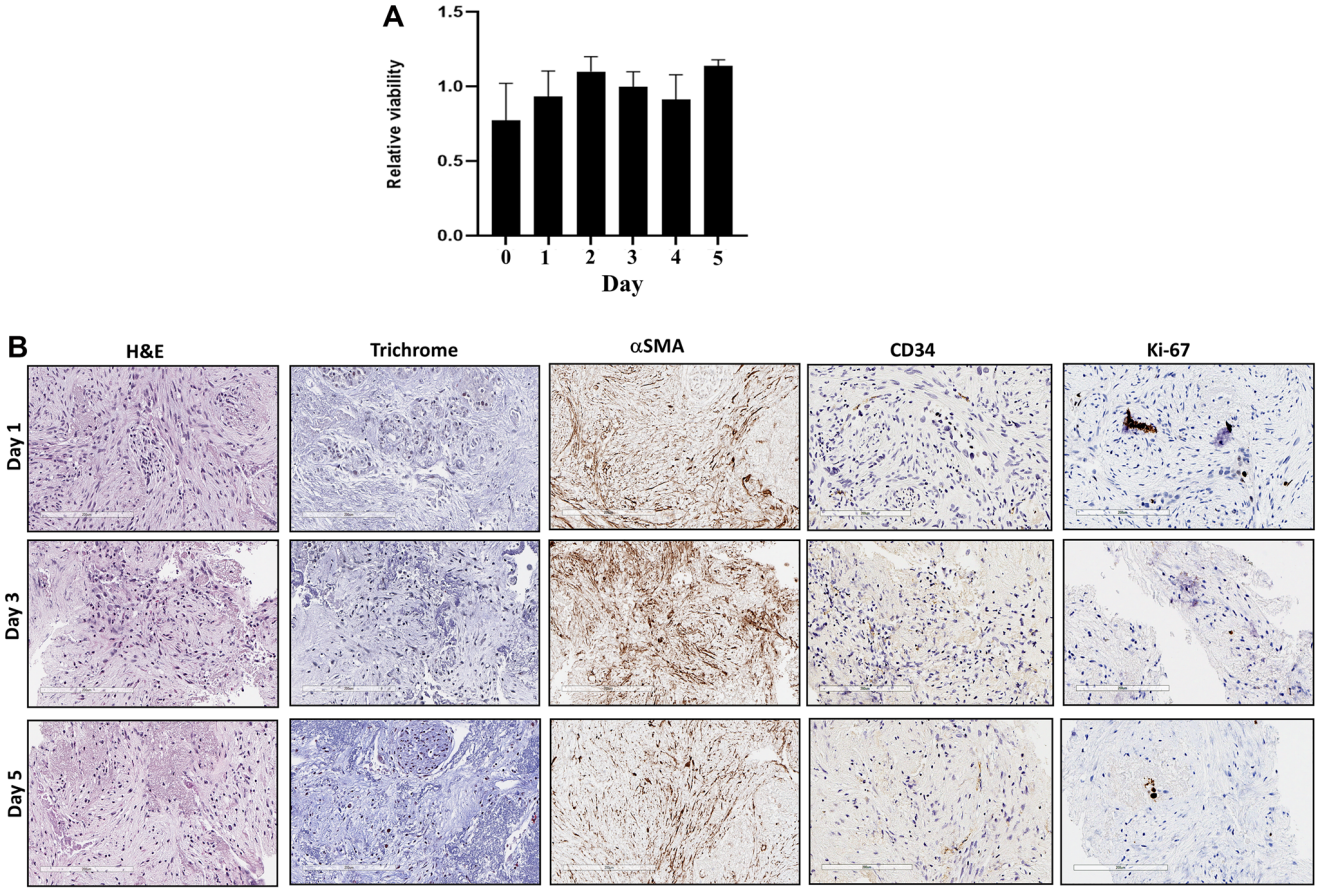
(**A**) Sliced fresh tumor samples were cultured in 96-well plates and viability was measured with PrestoBlue daily for 5 days. X-axis is days, and Y-axis is proportion of viability. (**B**) Sliced fresh tumor samples were embedded with paraffin, and tissue sections were stained with hematoxylin and eosin (H&E), Masson trichrome, αSMA, CD34 and Ki-67 antibodies.

**Figure 2:**
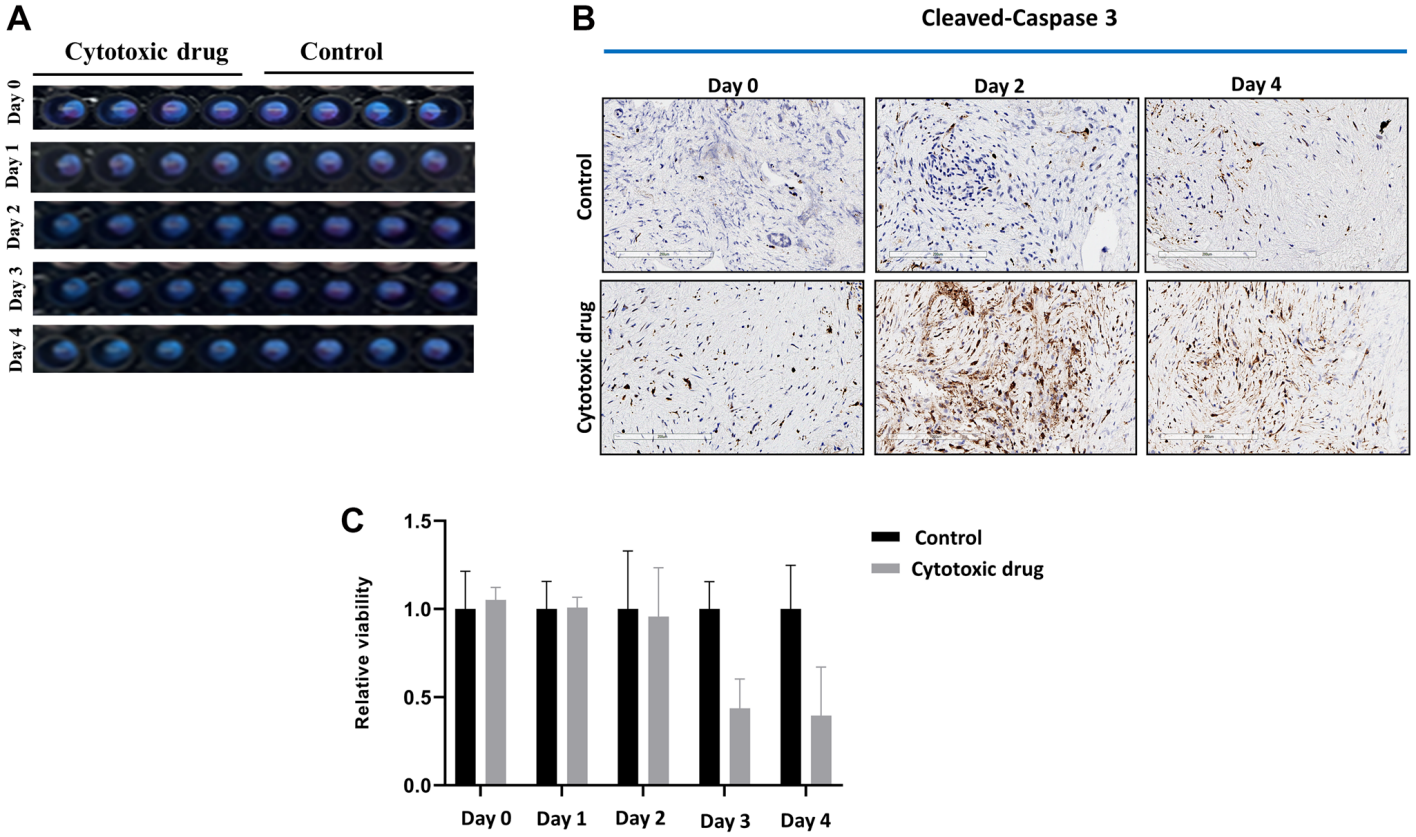
(**A**) Sliced fresh tumor samples were treated with or without cytotoxic drug (fluorouracil/SN-38(irinotecan)/oxaliplatin) in 96-well plates for 5 days. (**B**) Cytotoxic drug treated and untreated (control) sliced fresh tumor samples were immunohistochemically stained with anti-cleaved-caspase 3 antibody. (**C**) Viability of cytotoxic drug treated and untreated samples was measured by PrestoBlue.

To confirm the viabilities and maintained tumor microenvironment of fresh human tumor slices for a 5-day culture period of RT-LTSA, the tissue slices were cultured in 96-well plates with medium. Over 90% of the original viability was observed for 5 days (Figure 1A). Tumor microenvironment including stroma/collagen (α-SMA and trichrome staining) and vascular endothelial cells (CD34) was preserved in *ex vivo* tissue slice culture over 5 days (Figure 1B). We observed slightly decreased Ki-67 staining on Day 5 (Figure 1B). Auranofin was used as a positive control to validate viability assay of RT-LTSA, and significantly decreased viability was observed after treatment of auranofin (Supplementary Figure 1). To determine optimal duration of drug treatment in RT-LTSA, tissue samples were treated with cytotoxic drugs (fluorouracil (5FU)/SN-38(irinotecan)/oxaliplatin) (Figure 2A), and viability was measured by immunohistochemical staining of cleaved-caspase 3 (Figure 2B), and PrestoBlue assay (Figure 2C). We observed significant cleaved-caspase 3 expression on Day 2 and significantly decreased viability of drug treated tissue slices compared with untreated control tissue slices on Day 3 (Figure 2B and 2C). Based on these findings, we decided to treat tumor samples with cytotoxic drugs for 3 days and then analyze viability.

### Correlation between PDX-based LTSA (PDX-LTSA) or cell line and RT-LTSA

**Figure 3:**
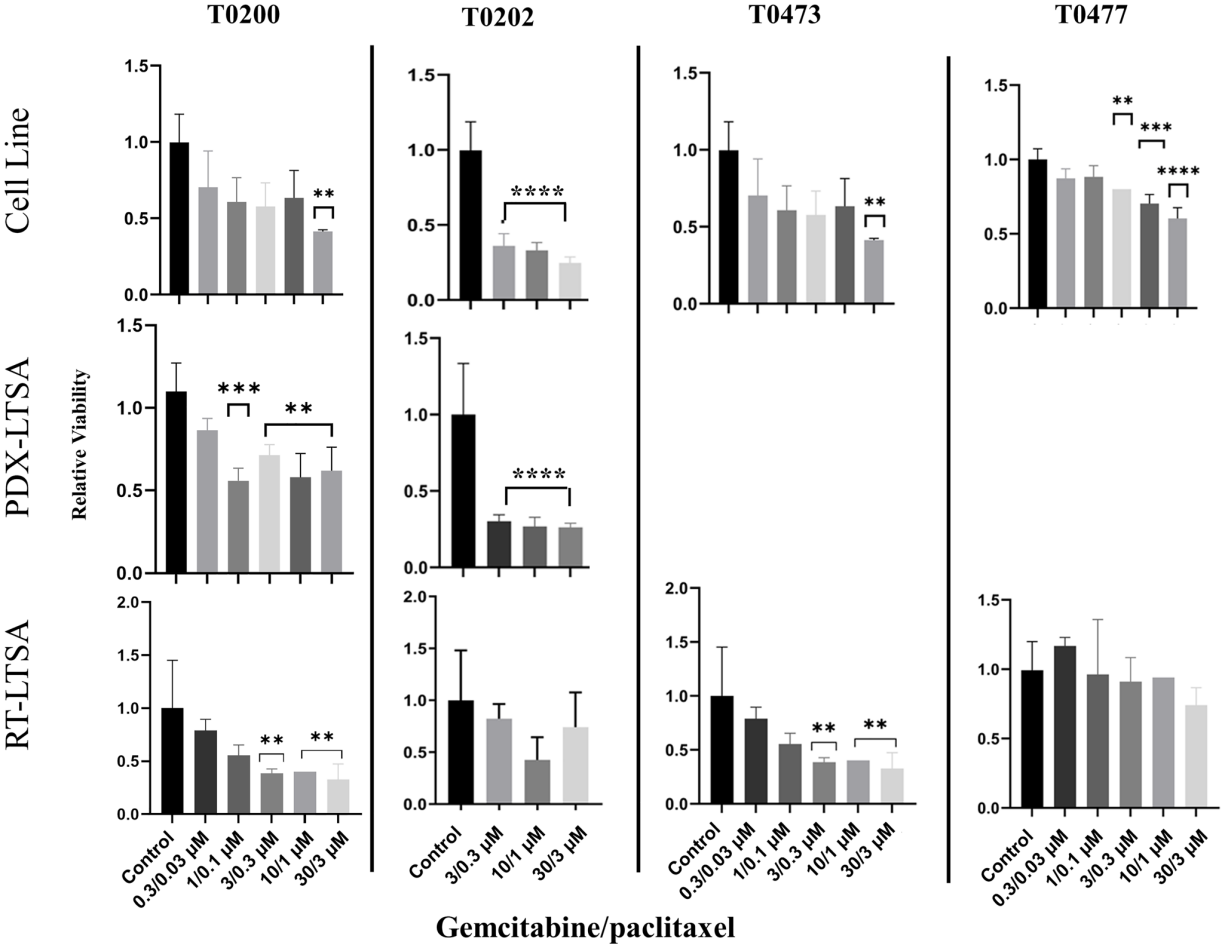
Tissue slices from the fresh tumor samples and the same origin patient-derived xenograft (PDX) and a cell line established from the same origin tumor were treated with gemcitabine and paclitaxel for 72 hours, and viability was measured with PrestoBlue. Abbreviations: LTSA: live tissue sensitivity assay; RT: real time. ^**^ *P* < 0.01, ^***^ *P* < 0.001, ^****^ *P* < 0.0001.

We reported that PDX-LTSA was reliable and predicted clinical response to therapeutic agents in patients with pancreatic adenocarcinoma, previously [5]. To evaluate the consistency of the result of a cancer cell line, PDX-LTSA and RT-LTSA, a PDX and a cancer cell line were established with fresh tumor samples. Tumor tissue slices from PDX and/or the cell line were treated with drugs and sensitivity was evaluated. Cell line test, PDX-LTSA and RT-LTSA assay demonstrated similar sensitivity of the same origin tumor to gemcitabine/paclitaxel in 2 tumor samples (T0200 and T0473) (Figure 3). However, no correlation was observed in the other 2 samples (T0202 and T0477). These findings suggest RT-LTSA may or may not correlate with PDX-LTSA and chemo-sensitivity of cancer cell lines in part depending on tumor microenvironment.

### Correlation between RT-LTSA and clinical outcome

**Figure 4:**
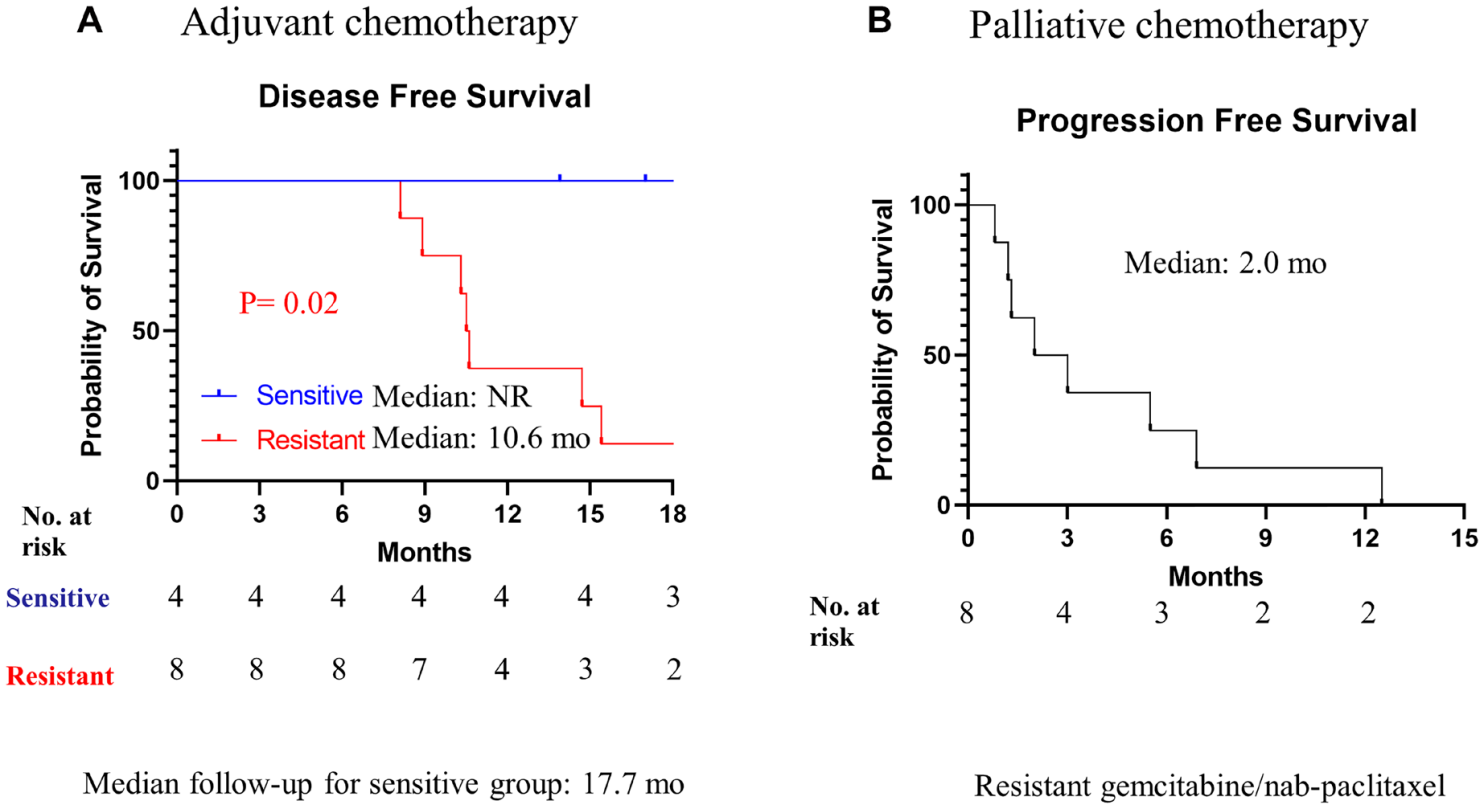
Kaplan-Meier analysis of disease-free survival and progression free survival overall survival. (**A**) Patients received RT-LTSA sensitivity adjuvant FOLFIRINOX (sensitive) or RT-LTSA resistant adjuvant FOLFIRINOX (resistant). (**B**) All patients received RT-LTSA resistant gemcitabine/nab-paclitaxel.

**Figure 5:**
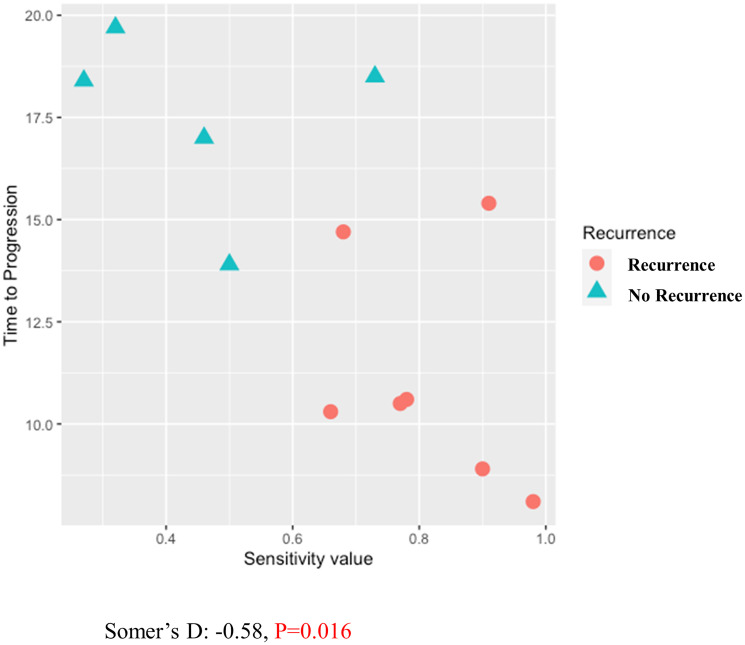
Correlation between RT-LTSA value and disease-free survival using Somer’s D statistics.

A total of 15 patients’ tumors were collected from surgically resected pancreatic cancer. The median age of the patients was 63 years (range 34–83), and 43% were male. While 4 patients received RT-LTSA sensitive adjuvant FOLFIRINOX chemotherapy, 8 received RT-LTSA resistant adjuvant FOLFIRINOX (*N* = 6) or gemcitabine/capecitabine (*N* = 2) (Table 1). While 7 of 8 patients who received RT-LTSA resistant adjuvant chemotherapy developed metastatic disease, none of 4 patients who received RT-LTSA sensitive adjuvant chemotherapy developed metastatic disease. We observed significantly prolonged disease-free survival in patients who received RT-LTSA sensitive adjuvant chemotherapy (median: no reached versus 10.6 months, *P* = 0.02) compared with the patient who received RT-LTSA resistant adjuvant chemotherapy respectively (Figure 4A). Median follow-up of the patients with RT-LTSA sensitive adjuvant chemotherapy was 17.7 months. One patient was found to develop liver metastasis during pancreatectomy, and biopsy samples of the liver metastasis were used for RT-LTSA. The patient with the liver metastasis received RT-LTSA sensitive FOLFIRINOX, and he achieved a partial response. Eight patients received RT-LTSA resistant gemcitabine/nab-paclitaxel when they developed metastatic disease. Their median progression free survival was 2.0 months (95% confidence interval: 0–4.4) (Figure 4B). Among the 8 patients who received RT-LTSA resistant gemcitabine/nab-paclitaxel for metastatic disease, 5 patients experienced disease progression, and 3 achieved stable disease as the best overall response. The correlation between RT-LTSA value and disease-free survival was evaluated, and a significant negative correlation (Somer’s D: −0.58; 95% confidence interval: −0.96 - −0.06; *P* = 0.016) was observed (Figure 5).

**Table 1:** Patients’ characteristics and clinical outcome

Age	Sex	TNM staging	Adjuvant chemotherapy	RT-LTSA sensitivity	DFS (months)	Sites of metastasis/recurrence	Palliative chemotherapy	RT-LTSA sensitivity	PFS (months)	OR
56	M	T2N1M0	FOLFIRINOX	Resistant	14.7	Pancreas, liver	Gemcitabine/ nab-pacliataxel	Resistant	0.8	PD
53	F	T2N1M0	Gemcitabine/ capecitabine	Resistant	8.9	Peritoneum	Gemcitabine/ nab-pacliataxel	Resistant	1.2	PD
65	F	T2N1M0	FOLFIRINOX	Resistant	10.6	Lung	Gemcitabine/ nab-pacliataxel	Resistant	6.9	SD
83	F	T2N1M0	None	NA	NA	Liver	Gemcitabine/ nab-pacliataxel	Resistant	5.5	SD
34	F	T1N0M0	FOLFIRINOX	Resistant	15.4	Liver	Gemcitabine/ nab-pacliataxel plus microwave ablation	Resistant	12.5	SD
80	M	T2N2M0	Gemcitabine/ capecitabine	Resistant	8.1	Lung	Gemcitabine/ nab-pacliataxel	Resistant	2	PD
54	F	T2N1M0	None	NA	NA	Peritoneum	Gemcitabine/ nab-pacliataxel	Resistant	1.3	PD
71	M	T1N0M0	FOLFIRINOX	Resistant	10.5	Peritoneum	Liposomal irinotenca/ 5FU	NA	4.7+	SD
70	M	T2N0M0	FOLFIRINOX	Resistant	10.3	Liver	Gemcitabine/ nab-pacliataxel	Resistant	3	PD
61	M	T2N0M0	FOLFIRINOX	Resistant	18.5+	NA	none	NA	NA	NA
45	F	T2N0M0	FOLFIRINOX	Sensitive	19.7+	NA	none	NA	NA	NA
67	F	T2N0M0	FOLFIRINOX	Sensitive	18.4+	NA	none	NA	NA	NA
63	F	T2N2M0	FOLFIRINOX	Sensitive	13.9+	NA	none	NA	NA	NA
69	M	T1N1M0	FOLFIRINOX	Sensitive	17.0+	NA	none	NA	NA	NA
62	M	T1N1M1	None	NA	NA	Liver	FOLFIRINOX	Sensitive	12	PR

Abbreviations: +: ongoing response; DFS: disease free survival; NA: not available; OR: objective response; PD: progressive disease; PFS: progression free survival; PR: partial response; RT-LTSA: real time-live tissue assay; SD: stable disease.

## DISCUSSION

Several methods and techniques including PDX and patient-derived cancer organoids (PDO) have been developed for drug-screening applications, and these approaches have demonstrated early promise in personalized therapy [6, 7]. Previously, we reported that PDX-LTSA could reflect clinical response, and it could be used as a personalized strategy to improve clinical outcome of pancreatic cancer [5]. However, current PDO- or PDX-based assays have significant limitations for the real time personalized strategy in pancreatic cancer since (1) developing a PDO or PDX model generally requires 2–3 months for PDO [6] or 4–8 months for PDX [8], which is close to median progression free survival or overall survival of patients with metastatic pancreatic cancer [4], (2) tumor-associated stroma and fibroblasts of origin tumor are lost in PDO and replaced by murine-derived extracellular matrix in PDXs [9], which may alter phenotype and drug sensitivity of origin tumor, and (3) the PDO and PDX engraftment failure rates are not insignificant (30–50%) in pancreatic cancer [7, 8]. In this study, we developed fresh tissue-based real time LTSA (RT-LTSA) to overcome these challenges of PDO- or PDX-based assays. The RT-LTSA could be completed within 3 days for evaluation of chemosensitivity after acquiring fresh samples, and it can be potentially applied to clinical practice to select the most effective chemotherapy regimen for patients immediately after tumor sample collection. Our data showed collected tumor samples remained viable for 5 days, and human tumor microenvironment/architecture including stroma/collagen (SMA and trichrome staining) and vascular endothelial cells (CD34 staining) were well-preserved for 5 days (Figure 1). We observed the significant negative correlation between RT-LTSA value and relapse-free survival (*P* = 0.016) (Figure 5), and none of the patients who received RT-LTSA sensitive chemotherapy developed recurrent or metastatic pancreatic cancer with the median follow-up of 17.7 months. In addition, the median disease-free survival (10.6 months) and median progression-free survival (2.0 months) of patients who received RT-LTSA resistant chemotherapy are shorter than historical data which are 21.6 months of median disease-free survival and 5.5 months of median progression-free survival [4, 10]. These data suggest our RT-LTSA may reflect clinical outcome effectively. We also observed significantly prolonged disease-free survival in patients who received RT-LTSA sensitive adjuvant chemotherapy compared with the patient who received RT-LTSA resistant adjuvant chemotherapy (*P* = 0.02) (Figure 4A).

There are several limitations of our study. Our approach needs sufficient tumor samples. Since pancreatic adenocarcinoma is associated with extensive desmoplasia and fibrosis, and stromal component can outnumber cancer cells [11], RT-LTSA is a highly dependent tumor cellularity of tumor samples. We think most of surgically resected samples can use RT-LTSA unless it has significant necrosis from previous treatment. However, our approach may not be ideal for locally advanced pancreatic cancer or low tumor burden disease, and extensive drug screening may not be feasible depending on the size of tumor samples.

Our data showed significant discrepancy of chemosensitivity between RT-LTSA and cell line/PDX assay in 4 different tumor samples (Figure 3). Some of tumor samples showed the correlation of RT-LTSA and cell line/PDX assay but the others did not. This can be explained by the fact that tumor microenvironment including caner associated fibroblasts, pancreatic stellate cells, extracellular matrix and infiltrated immune cells may play a unique role in chemoresistance to pancreatic cancer in addition to tumor cells [12].

In summary, we report the establishment of fresh tumor tissue-based RT-LTSA which may maintain intact human tumor microenvironment and architectures and be predictive of clinical response in pancreatic adenocarcinoma within 3 days. This approach may allow clinicians to select the most effective therapeutic agents with real time in patients with pancreatic adenocarcinoma. Further prospective studies are warranted to verify our findings and clinical application.

## MATERIALS AND METHODS

### Patient selection and clinical data collection

Under an Institutional Review Board-approved protocol, fresh biospecimens were collected from consented patients undergoing pancreas resection for pancreatic ductal adenocarcinoma. Patient demographics, primary tumor characteristics, treatment and clinical outcomes were collected.

### *Ex vivo* tissue slice culture and drug treatment

Fresh resected tumor samples were obtained in operating room from consented patients. Tissue cores were generated with 3-mm disposable biopsy punches (Integra Miltex, York, PA) from resected tumor tissues and immediately put in Belzer UW^®^ Cold Storage Solution supplemented with 2% Penicillin-Streptomycin-Neomycin (PSN) antibiotic mixture. Tissue cores were embedded in 1% low melting-point agarose gel (Sigma, St. Louis, MO) and cut into slices (200 μm) with the Krumdieck Tissue Slicer (Alabama Research and Development, Munford, AL). With this technique, depending on the number of cores obtained, approximately 100–150 tissue slices were generated. The tissue slices were randomly arrayed in 96-well plates with 100 μl RPMI1640 medium supplemented with 10% fetal bovine serum (FBS) and 2% PSN and incubated in a humidified 37°C incubator supplied with 5% CO_2_. The plates were seated on a platform shaker at 150 RPM. After 2 hours incubation, tissue slices were treated with gemcitabine (0.3 μM, 1 μM, 3 μM, 30 μM, 100 μM), paclitaxel (0.03 μM, 0.1 μM, 0.3 μM, 1 μM, 3 μM), fluorouracil (0.3 μM, 1 μM, 3 μM, 10 μM, 30 μM), SN-38 (irinotecan: 0.03 μM, 0.1 μM, 0.3 μM, 1 μM, 3 μM) and oxaliplatin (0.03 μM, 0.1 μM, 0.3 μM, 1 μM, 3 μM) in an additional 100 μl medium, totaling 200 μl medium per well. Auranofin (10 μM) was used as a positive control. The plates were returned to the incubator/shaker and cultured for 24–72 hours. Pancreatic cancer cell lines and tumor xenografts were established from patients as described previously [13].

### Tissue slice viability assay

After the treatment period, 20 μl of 10X Prestoblue^®^ reagent was added to the tissue slice culture medium, and the plates were incubated for an additional 2 hours on the shaker. Tissue slice viabilities were measured through reading fluorescence intensity with a CLARIOstar^®^ plate reader (BMG LABTECH, Cary, NC) and were normalized against the viability of control (untreated) slices.

### Masson’s trichrome and immunohistochemical staining

Tissue slices were fixed with 10% formalin for 2 hours followed by embedding in paraffin. Embedded tissue slices were cut into 5 μm sections. Masson’s trichrome stain was performed according to the manufacturer’s instructions (Trichrome Stain Masson Kit; Sigma-Aldrich, St. Louis. MO). Immunohistochemistry staining was performed with Lab-Vision 480-2D immunostainer (Thermo Fisher, Fremont, CA). αSMA (Abcam, Cambridge, MA), CD34 (Abcam, Cambridge, MA), Ki-67 (Cell Signaling, Danvers, MA) and cleaved-caspase 3 (Cell Signaling, Danvers, MA) antibodies were used for immunohistochemical staining with the manufacturer’s instructions.

### Statistical methods

The significance of differences in tissue slice viabilities between treatment and no-treatment groups was analyzed by Student *t*-test (two tails). Cut-off value for defining the sensitivity in RT-LTSA assay was determined with receiver operating characteristic (ROC) curve analysis. We used the receiver operating characteristic curve analysis of LTSA value from the highest dose of gemcitabine, and identified that a value of 0.68 is the optimal cut-off (when AUC = 1) to define the sensitivity (LTSA value ≤0.68) or resistance (LTSA value >0.68) of tissue slices to the treatment from our previous data [5]. Correlation of RT-LTSA value and disease-free survival was analyzed with Somer’s D statistics since several patients have ongoing response (censored). The significance of the clinical outcome data was determined by Mann-Whitney *U* test (two tailed). All statistical analyses were performed using IBM SPSS Statistics 24. All statistical tests used a significance level of 5%. No adjustments for multiple testing were made.

## SUPPLEMENTARY MATERIALS



## References

[1] Siegel RL , Miller KD , Wagle NS , Jemal A . Cancer statistics, 2023. CA Cancer J Clin. 2023; 73:17–48. 10.3322/caac.21763. 36633525

[2] Lee JC , Ahn S , Cho IK , Lee J , Kim J , Hwang JH . Management of recurrent pancreatic cancer after surgical resection: a protocol for systematic review, evidence mapping and meta-analysis. BMJ Open. 2018; 8:e017249. 10.1136/bmjopen-2017-017249. 29632079 PMC5892773

[3] Conroy T , Desseigne F , Ychou M , Bouché O , Guimbaud R , Bécouarn Y , Adenis A , Raoul JL , Gourgou-Bourgade S , de la Fouchardière C , Bennouna J , Bachet JB , Khemissa-Akouz F , et al, and Groupe Tumeurs Digestives of Unicancer, and PRODIGE Intergroup. FOLFIRINOX versus gemcitabine for metastatic pancreatic cancer. N Engl J Med. 2011; 364:1817–25. 10.1056/NEJMoa1011923. 21561347

[4] Von Hoff DD , Ervin T , Arena FP , Chiorean EG , Infante J , Moore M , Seay T , Tjulandin SA , Ma WW , Saleh MN , Harris M , Reni M , Dowden S , et al. Increased survival in pancreatic cancer with nab-paclitaxel plus gemcitabine. N Engl J Med. 2013; 369:1691–703. 10.1056/NEJMoa1304369. 24131140 PMC4631139

[5] Roife D , Dai B , Kang Y , Perez MVR , Pratt M , Li X , Fleming JB . Ex Vivo Testing of Patient-Derived Xenografts Mirrors the Clinical Outcome of Patients with Pancreatic Ductal Adenocarcinoma. Clin Cancer Res. 2016; 22:6021–30. 10.1158/1078-0432.CCR-15-2936. 27259561 PMC5136340

[6] Driehuis E , van Hoeck A , Moore K , Kolders S , Francies HE , Gulersonmez MC , Stigter ECA , Burgering B , Geurts V , Gracanin A , Bounova G , Morsink FH , Vries R , et al. Pancreatic cancer organoids recapitulate disease and allow personalized drug screening. Proc Natl Acad Sci U S A. 2019; 116:26580–90. 10.1073/pnas.1911273116. 31818951 PMC6936689

[7] Pham NA , Radulovich N , Ibrahimov E , Martins-Filho SN , Li Q , Pintilie M , Weiss J , Raghavan V , Cabanero M , Denroche RE , Wilson JM , Metran-Nascente C , Borgida A , et al. Patient-derived tumor xenograft and organoid models established from resected pancreatic, duodenal and biliary cancers. Sci Rep. 2021; 11:10619. 10.1038/s41598-021-90049-1. 34011980 PMC8134568

[8] Izumchenko E , Paz K , Ciznadija D , Sloma I , Katz A , Vasquez-Dunddel D , Ben-Zvi I , Stebbing J , McGuire W , Harris W , Maki R , Gaya A , Bedi A , et al. Patient-derived xenografts effectively capture responses to oncology therapy in a heterogeneous cohort of patients with solid tumors. Ann Oncol. 2017; 28:2595–605. 10.1093/annonc/mdx416. 28945830 PMC5834154

[9] Chao C , Widen SG , Wood TG , Zatarain JR , Johnson P , Gajjar A , Gomez G , Qiu S , Thompson J , Spratt H , Hellmich MR . Patient-derived Xenografts from Colorectal Carcinoma: A Temporal and Hierarchical Study of Murine Stromal Cell Replacement. Anticancer Res. 2017; 37:3405–12. 10.21873/anticanres.11707. 28668828 PMC5548438

[10] Conroy T , Hammel P , Hebbar M , Ben Abdelghani M , Wei AC , Raoul JL , Choné L , Francois E , Artru P , Biagi JJ , Lecomte T , Assenat E , Faroux R , et al, and Canadian Cancer Trials Group and the Unicancer-GI–PRODIGE Group. FOLFIRINOX or Gemcitabine as Adjuvant Therapy for Pancreatic Cancer. N Engl J Med. 2018; 379:2395–406. 10.1056/NEJMoa1809775. 30575490

[11] Feig C , Gopinathan A , Neesse A , Chan DS , Cook N , Tuveson DA . The pancreas cancer microenvironment. Clin Cancer Res. 2012; 18:4266–76. 10.1158/1078-0432.CCR-11-3114. 22896693 PMC3442232

[12] Wang S , Li Y , Xing C , Ding C , Zhang H , Chen L , You L , Dai M , Zhao Y . Tumor microenvironment in chemoresistance, metastasis and immunotherapy of pancreatic cancer. Am J Cancer Res. 2020; 10:1937–53. 32774994 PMC7407356

[13] Kim MP , Evans DB , Wang H , Abbruzzese JL , Fleming JB , Gallick GE . Generation of orthotopic and heterotopic human pancreatic cancer xenografts in immunodeficient mice. Nat Protoc. 2009; 4:1670–80. 10.1038/nprot.2009.171. 19876027 PMC4203372

