# Correction: Real time *ex vivo* chemosensitivity assay for pancreatic adenocarcinoma

**DOI:** 10.18632/oncotarget.28556

**Published:** 2024-07-02

**Authors:** Dae Won Kim, Francisca Beato, Youngchul Kim, Alexandra F. Tassielli, Ruifan Dai, Jason W. Denbo, Pamela J. Hodul, Mokenge P. Malafa, Jason B. Fleming

**Affiliations:** ^1^Department of Gastrointestinal Oncology, Moffitt Cancer Center, Tampa, FL 33612, USA; ^2^Department of Biostatistics and Bioinformatics, Moffitt Cancer Center, Tampa, FL 33612, USA; ^*^These authors contributed equally to this work

**This article has been corrected:** The following author has been added to this paper.

Bhaswati Sarcar

Department of Gastrointestinal Oncology, Moffitt Cancer Center, Tampa, FL 33612, USA

Original article: Oncotarget. 2023; 14:811–818. 811-818. https://doi.org/10.18632/oncotarget.28508

