# Supplementary material for: Real time ex vivo chemosensitivity assay for pancreatic adenocarcinoma
Source: Oncotarget. 2023 Sep 15;14:811–8. doi: 10.18632/oncotarget.28508 (PMC10503742; doi:10.18632/oncotarget.28508)
Supplement: Supplementary file 1 [file oncotarget-14-28508-s001.pdf]

Real time *ex vivo* chemosensitivity assay for pancreatic adenocarcinoma

SUPPLEMENTARY MATERIALS

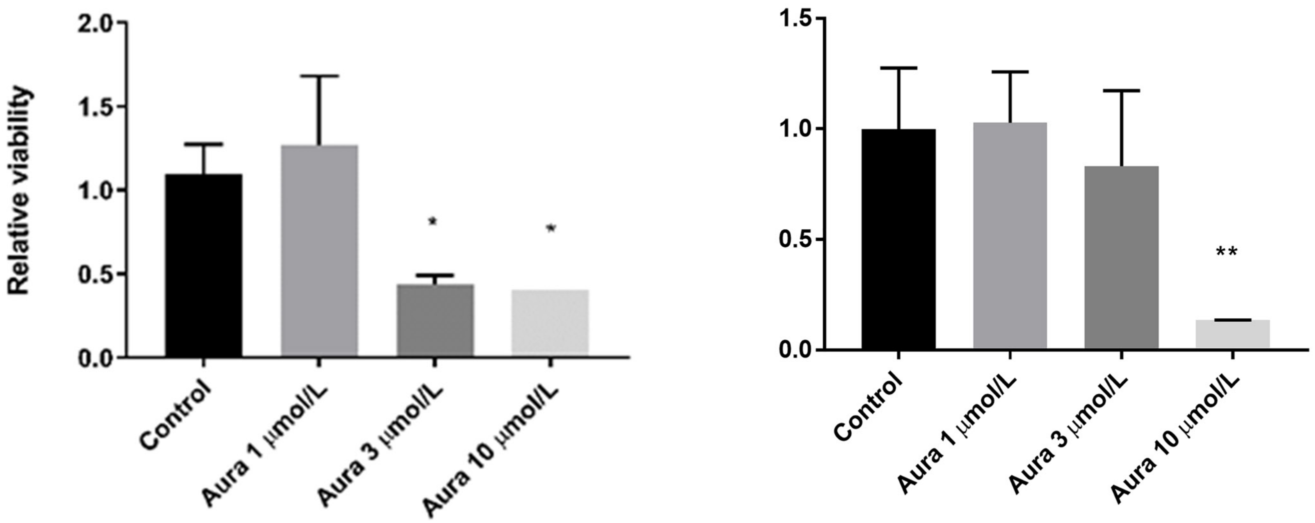

Supplementary Figure 1: Tissue slices from two different fresh tumor samples were treated with auranofin, and viability was measured with PrestoBlue. $^{*}P < 0.05$, $^{**}P < 0.01$.
